# Migration of the distal catheter of the ventriculoperitoneal shunt in hydrocephalus patients

**DOI:** 10.17712/nsj.2017.4.20170137

**Published:** 2017-10

**Authors:** Mohammed Z. Allouh, Mohammed M. Al Barbarawi, Mohammed H. Hiasat, Bashar A. Abuzayed

**Affiliations:** *From the Department of Anatomy (Allouh), Division of Neurosurgery (Al Barbarawi, Hiasat), Department of Neuroscience, Faculty of Medicine, Jordan University of Science & Technology, and from Division of Neurosurgery (Abuzayed), Department of Neuroscience, King Abdullah University Hospital, Irbid, Jordan*

## Abstract

Distal catheter migration of a ventriculoperitoneal shunt (VPS) is a rare but serious complication. It is usually asymptomatic. However, it can be further complicated by the presence of co-infections, interruption of the shunt, and/or disturbances in penetrated organ function. In this report, we presented a case of spontaneous complete extrusion of the distal end of a VPS catheter through the intact abdominal wall in a 5-year-old boy with hydrocephalus. We also reviewed and analyzed the literature for similar cases of complete extrusion of the distal end of a VPS catheter, through an intact or a potential weakness in the body wall, in the last 20 years. From the reviewed literature, we did not observe any difference (p>0.05) in the incidence of this complication between cases with an intact or a potential weakness in the body wall.

Ventriculoperitoneal shunt (VPS) installation is a widely used method in hydrocephalus treatment. However, it is associated with a number of complications relating to the proximal and distal ends of the shunt, with complications at the distal end occurring more frequently.^1^ Distal abdominal complications may include obstruction, disconnection, pseudocyst formation, peritonitis, hydroceles, and catheter migration. Kanojia et al^2^ reported that the incidence of distal VPS migration accounts for approximately 10% of all VPS complications. Distal VPS migration can have different presentations. The catheter may penetrate through hollow viscera (e.g., the heart, intestine, stomach, and urinary bladder). It may also penetrate through an intact or a potential weakness (e.g., the inguinal canal or umbilicus) in the abdominal wall, or through the gastrointestinal tract to exit either transorally or transanally.^1^ In general, distal VPS migration usually remains asymptomatic for a prolonged period. However, it may be exacerbated by the presence of co-infections that can be life threatening. The most common complications associated with distal VPS migration are meningitis and ventriculitis.^1^,^2^

Herein, we present a case of spontaneous extrusion of the distal VPS catheter through the intact abdominal wall at an area unrelated to the surgical incision. Both clinical and radiological findings along with the treatment regimens are discussed. In addition, we reviewed similar cases in the literature in order to improve the understanding and management of this complication.

## Case Report

### History and presentation

**Figure 1:**
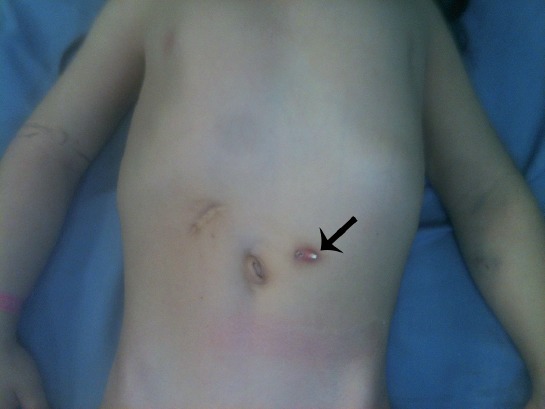
Extrusion of the distal end of a ventriculoperitoneal shunt catheter (arrow tip) through the intact abdominal wall in a 5-year-old male with Apert syndrome.

A 5-year-old boy, with a previous VPS insertion and Apert syndrome, presented with spontaneous complete extrusion of the distal end of a VPS catheter through the intact abdominal wall (**Figure 1**). At 1-month of age, the patient underwent a VPS installation for congenital hydrocephalus. His medical record showed no previous shunt revisions were conducted until presentation. On admission, he was conscious and had no neurological deficits. Physical examination revealed an old scar in the right subcostal region due to the previous VPS insertion. The distal peritoneal catheter protruded from the left paraumbilical region of the abdominal wall.

### Laboratory findings and treatment intervention

**Figure 2:**
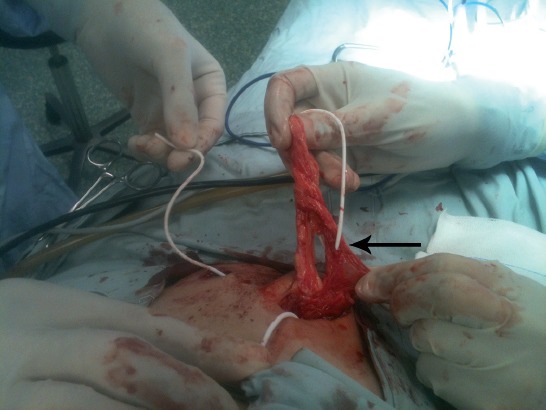
Laparotomy procedure for the detachment of a migrated distal ventriculoperitoneal shunt catheter (arrow tip) from the greater omentum in a 5-year-old male with Apert syndrome.

Laboratory findings were essentially within normal limits. The cerebrospinal fluid (CSF) culture was negative and an abdominal computed tomography (CT) scan did not reveal any intra-abdominal pathology. A prophylactic antibiotic regimen that consisted of ceftriaxone (50 mg/kg twice daily) and vancomycin (15 mg/kg twice daily) was commenced 24 hours prior to surgical replacement of the shunt. The entire shunt system was replaced with a Strata^®^ VPS. The distal peritoneal catheter was found to be adherent to the greater omentum and a small laparotomy was necessary by the pediatric surgeon to detach it (**Figure 2**).

### Postoperative course

He had an uneventful postoperative course. Prophylactic antibiotic treatment was continued for the duration of the hospital stay. Serial postoperative CSF cultures were negative and a postoperative CT scan demonstrated a reduction in ventricular size. He was discharged after 8 days with scheduled follow-up appointments. At 6-months follow-up, he was doing well.

## Discussion

Despite being a serious condition, the exact cause of distal VPS migration is usually not determined. However, several hypotheses have been proposed. Akyüz et al^3^ reported that when the distal end of the catheter adheres to a nearby viscera or body wall, it will initiate an inflammatory response that weakens the viscera or body wall, and the distal end of the catheter will gradually protrude through it. Sridhar et al^4^ suggested that distal VPS migration may occur due to the firmness of the type of catheter used. This hypothesis is supported by the observation that there is reduced occurrence of distal VPS migration when using softer, more flexible catheters.^2^ In addition, other authors have speculated that distal VPS extrusion through the body wall may occur due to focal wound dehiscence, poor host immunity, inappropriate surgical technique, or ischemic necrosis of the dermis.^5^,^6^

Additional contributing factors for distal VPS migration may include the patients’ age, as well as the length of the distal catheter inside the peritoneal cavity. For example, we noticed that most cases of distal VPS migration described in the literature have occurred in early childhood. This may be attributed to the weak musculature at this period of life that may ease penetration of the catheter through the viscera or body wall. In addition, we speculate that a longer catheter may have a higher tendency to migrate than a shorter one. However, further studies are required to confirm these hypotheses.

When spontaneous extrusion of the distal catheter of a VPS through the abdominal wall is detected, a course of prophylactic antibiotics should be administered immediately and the shunt system must be replaced completely.^5^,^7^ During removal of the extruded shunt system, it is important to avoid pulling the distal end proximally in order to prevent the spread of infection from the extrusion site. If there is no peritoneal or skin infection then the VPS catheter can be removed without laparotomy. Laparotomy is indispensable in the presence of peritonitis and in cases where the catheter has adhered to components of the peritoneum. After complete removal of the existing shunt system, reinsertion of a new shunt system may be performed in the same session, provided that the CSF culture was negative. However, if an infection is suspected then an external ventricular drainage system is placed and an intravenous course of antibiotics is administered.^8^,^9^ Serial CSF sampling is subsequently carried out until 2 consecutive, negative culture results are obtained.^2^ Finally, reinsertion of a new shunt system can be performed. A review of the literature for cases of complete catheter extrusion in the last 20 years identified 24 studies reported on 31 cases of external distal VPS migration. Detailed information regarding these cases is provided in **Table 1**. Of these 31 cases, 14 (~45%) had the distal catheter extruded through the intact body wall, while the remaining 17 cases (~55%) had the catheter extruded through a potential weakness in the body wall. A summary for the frequencies of different extrusion types is provided in **Table 2**. No significant difference in the incidence of distal VPS catheter extrusion was observed between cases through an intact body wall and cases through potential weakness in the body wall (*p*=0.59, chi-square goodness of fit test). This may indicate that the presence of a potential weakness in the body wall is not a predisposing factor for external migration of the distal VPS catheter.

**Table 1:** Literature review of cases of complete extrusion of the distal end of a ventriculoperitoneal shunt catheter in the last 20 years.

References	Cases (n)	Extrusion type	Extrusion site	Age at presentation (yrs)	Post-insertion duration (yrs)
Nourisamie et al,^10^ 2001	1	Intact	Left thigh	<1 (10 mo)	<1 (2 mo)
Pandey et al,^11^ 2003	1	Intact	Left posterior auricular region	10.5	<1 (6 mo)
Schulz and Labram,^12^ 2006	1	Intact	Epigastric region	26	14
Kanojia et al,^2^ 2008	4	Intact	Right lumbar region	<1 (3-6 mo)	<1 (1-3 mo)
		Intact	Cervical region		
		Intact	Cervical region		
		Weakness	Umbilical region		
Borkar et al,^5^ 2008	1	Intact	Right anterior chest wall	14	3
Vural et al,^13^ 2008	1	Intact	Sacrococcygeal region	<1 (7 mo)	<1 (7 mo)
Birbilis et al,^8^ 2009	1	Intact	Left paraumbilical region	33	1
Silva Neto et al,^9^ 2011	1	Intact	Right posterior abdominal wall	5	5
Dağtekin et al,^14^ 2011	1	Intact	Umbilical region	2	1.7
Panigrahi et al,^15^ 2012	2	Intact	Epigastrium	<1 (7 mo)	<1 (3 mo)
		Intact	Epigastrium	14	8
Oktay et al,^16^ 2015	1	Intact	Right lumbar region	1	1
Rehm et al,^17^ 1997	1	Weakness	Scrotum	46	4
Esposito et al,^18^ 1998	1	Weakness	Umbilicus	14	N/A
Wani et al,^19^ 2002	1	Weakness	Umbilicus	1.5	<1 (6 mo)
Silav et al,^20^ 2002	1	Weakness	Umbilicus	N/A	N/A
Chan et al,^21^ 2003	1	Weakness	Left lumbar wound scar	70	6
de Aquino et al,^22^ 2006	1	Weakness	Umbilicus	1.6	N/A
Eser et al,^23^ 2006	1	Weakness	Umbilicus	<1 (3 mo)	<1 (3 mo)
Kella et al,^24^ 2008	1	Weakness	Umbilicus	1.5	1.4
Kumar et al,^6^ 2010	1	Weakness	Umbilicus	<1 (3 mo)	<1 (3 mo)
De Jong et al,^7^ 2011	1	Weakness	Umbilicus	38	38
Ghritlaharey et al,^25^ 2012	4	Weakness	Neck wound scar	<12	1.4
		Weakness	Anterior chest wall wound scar		2
		Weakness	Upper right abdomen wound scar		<1 (2 mo)
		Weakness	Umbilicus		<1 (3 mo)
Aras et al,^26^ 2013	1	Weakness	Posterior abdominal wall wound scar	1.8	<1 (8 mo)
Fleissig et al,^27^ 2013	1	Weakness	Umbilicus	82	2.3

N/A - not available, yrs - years, mo – months

**Table 2:** Frequency of extrusion of the distal catheter of a ventriculoperitoneal shunt in hydrocephalus patients over the last 20 years.

Extrusion type	n (%)
Intact	14 (45.2)
***Weakness***	17 (54.8)
Umbilical	11 (35.5)
Wound scar	5 (16.1)
Inguinal	1 (3.2)

**Total**	**31 (100)**

In conclusion, migration of the distal VPS catheter is a rare but serious complication that is associated with a high morbidity and mortality rate. Proper management of distal VPS migration should include a course of prophylactic antibiotics and complete replacement of the shunt system, with laparotomy, if required (peritonitis or adhesion). In addition, strict follow-up should be performed for serial CSF cultures and in order to ensure correct functioning of the new shunt system. This study suggests that the presence of a potential weakness in the body wall may not be a predisposing factor for VPS catheter extrusion.
